# Can COVID-19 be a risk for cachexia for patients during intensive care? Narrative review and nutritional recommendations

**DOI:** 10.1017/S0007114520004420

**Published:** 2020-11-05

**Authors:** Isabel P. A. Virgens, Natália M. Santana, Severina C. V. C. Lima, Ana P. T. Fayh

**Affiliations:** 1 Graduate Program in Health Sciences, Federal University of Rio Grande do Norte, Natal, Rio Grande do Norte 59078-970, Brazil; 2 Graduate Program in Nutrition, Federal University of Rio Grande do Norte, Natal, Rio Grande do Norte 59078-970, Brazil

**Keywords:** Muscle wasting, Weight loss, Malnutrition, Coronavirus infections, Diet, COVID-19, coronavirus disease-2019, CT, computed tomography, ICU, intensive care unit, LOS, length of stay, SARS-CoV-2, severe acute respiratory syndrome coronavirus 2

## Abstract

Although increased weight, and particularly obesity, has been associated with a more severe clinical course of COVID-19 and risk of fatality, the course of the illness can lead to prolonged length of stay. Changes in nutritional status and weight loss during hospitalisation are largely reported in some populations, but still not explored in COVID-19 patients. Considering that patients with COVID-19 show an increased inflammatory response, other signs and symptoms, which can lead to weight and muscle loss, should be monitored. The aim of this article was to establish possible connections between COVID-19, prolonged hospitalisation and muscle wasting, as well as to propose nutritional recommendations for the prevention and treatment of cachexia, through a narrative review. Identification of risk and presence of malnutrition should be an early step in general assessment of all patients, with regard to more at-risk categories including older adults and individuals suffering from chronic and acute disease conditions, such as COVID-19. The deterioration of nutritional status, and consequently cachexia, increases the risk of mortality and needs to be treated with attention as other complications. There is, however, little hard evidence of nutritional approaches in assisting COVID-19 treatment or its management including cachexia.

## Graphical abstract

**Figure:**
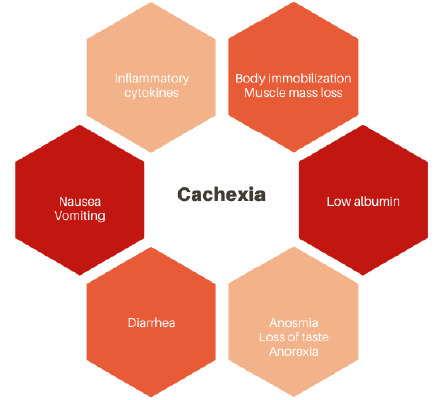

In December 2019, an outbreak of pneumonia cases of unknown cause was reported by health facilities in Wuhan, Hubei province, China in which clusters of patients were associated with the seafood and wet animal wholesale market^(1,2)^. On 7 January 2020, the novel coronavirus which caused the illness was identified in a throat swab sample^(2)^. On 11 February 2020, the WHO stated a new name for the malady: coronavirus disease-2019 (COVID-19)^(3)^ and 1 month later declared it as a global pandemic^(4)^. As stated by the WHO, until 6 September 2020, nearly 27 million cases of COVID-19 were reported of which 900 000 people died^(5)^.

COVID-19 is a respiratory disease caused by the novel coronavirus, severe acute respiratory syndrome coronavirus 2 (SARS-CoV-2), that has reached pandemic status^(6)^. Underlying diseases and other risk factors might contribute to the severity of COVID-19 patients. Chronic obstructive pulmonary disease, hypertension, diabetes, malnutrition, smoking habit, cerebrovascular disease, male sex, age over 65 years and obesity were reported to be associated with severe cases^(7–14)^. Although increased weight, and particularly obesity, has been associated with a more severe clinical course of COVID-19 and risk of fatality^(15,16)^, the course of the illness can lead to prolonged length of stay (LOS).

Changes in nutritional status and weight loss during hospitalisation are largely reported in some populations^(17,18)^, but still not explored in COVID-19 patients^(19)^. In fact, few studies reported nutritional status of patients during hospitalisation for COVID-19^(20)^. Symptoms and associated conditions might contribute to nutritional status deterioration, leading to cachexia^(21)^. Thus, the aim of this narrative review is to establish possible connections between COVID-19, prolonged hospitalisation and cachexia, and to propose nutritional recommendations for the prevention and treatment of such muscle wasting condition.

The literature review was conducted according to the SANRA Statement^(22)^ utilising PubMed, Lilacs, Google Scholar and Cochrane Library databases. First, to identify relevant publications about COVID-19 and cachexia, the combined search terms were used: (1) COVID-19 OR SARS-CoV-2, (2) cachexia OR muscle wasting and (3) diet OR nutrition. The inclusion criteria were studies published from January 2020 to 11 August 2020, published in English. Afterwards, to further discuss the relationship between COVID-19 infection, diet and loss of weight and muscle mass, relevant articles from the nutrition and cachexia area (including clinical characteristics and symptoms) were included.

## How can COVID-19 induce weight loss?

The virus SARS-CoV-2 is commonly transmitted through respiratory droplets, contact and potentially via the faecal–oral route^(23,24)^. First, viral replication occurs in the upper respiratory tract and subsequently reaches the lower respiratory tract and other tissues and organs, including the gastrointestinal tract^(24)^. In addition to respiratory symptoms, gastrointestinal symptoms caused by SARS-CoV-2 were also reported and they appear to exacerbate malnutrition in patients^(20)^. Therefore, COVID-19 was reported to be associated with malnutrition in some studies^(20,25,26)^. Diarrhoea, mild abdominal pain, nausea, vomiting, poor appetite and other symptoms were commonly reported and can cause reduction in food intake and/or absorption, and consequently weight loss^(27)^.

Another possible important implication to weight loss is the acute inflammatory chain in response to the SARS-CoV-2 infection. The virus has spike (S) proteins, a glycoprotein that has high affinity with the angiotensin-converting enzyme 2 receptor, which is the mediator of virus entry^(28,29)^. Angiotensin-converting enzyme 2 in the gastrointestinal tract has been identified in several studies, but it is widely expressed in various organs considered target for SARS-CoV-2 in humans, such as the nasal mucosa, bronchus, lung, heart, oesophagus, kidney and bladder^(24,30)^.

Once the virus entry occurs, the rapid viral replication and a series of reactions begin such as cellular damage, the cytokine storm and antibody-dependent enhancement^(24,30,31)^. The process referred above as ‘cytokine storm’ is characterised as the presentation of the viral antigens to the natural killer and CD8-positive cytotoxic T cells in the context of major tissue histocompatibility^(31)^.

After this event, it is possible that massive epithelial and endothelial cell death and vascular leakage happen, triggering the production of several pro-inflammatory cytokines and chemokines, which are responsible for the aggressive inflammation caused by SARS-CoV-2^(24,30)^. In this regard, acute phase proteins such as C-reactive protein, ferritin, TNF*α*, IL family factors, NF-*κ*B, interferon-*γ*, fibroblast growth factor and others are synthesised^(20)^. Antibody-dependent enhancement can promote interactions between virus-anti-S protein-neutralising antibodies and target cell receptors that can increase inflammatory response as well^(24)^.

All these processes are directly related to the increase of muscle proteolysis, albumin consumption and impaired metabolism of macronutrients which can contribute to the onset of malnutrition and cachexia^(30)^. A cross-sectional study evaluating malnutrition in 182 COVID-19 hospitalised elderly patients (mean age 68·5 (sd 8·8) years) in China found that subjects classified as malnourished showed significantly lower albumin levels and calf circumference^(20)^. However, changes in fat deposits and age-related loss of skin elasticity may contribute to errors in estimating muscle mass in the elderly^(32)^.

Considering that patients with COVID-19 show an increased inflammatory response upon hospital admission^(14)^, other signs and symptoms which can lead to weight and muscle loss should be monitored. Table 1 shows some studies in which symptoms may contribute to weight loss in COVID-19 patients. Notably, there is lack of information regarding some symptoms in these reports. It is also important to emphasise the lack of information regarding nutritional status, including BMI and weight loss in these studies. BMI was reported in a few studies in COVID-19 patients, although this marker of nutritional status has a significant association with the illness severity^(15,39–41)^. Older age and the presence of co-morbid conditions are almost invariably associated with impaired nutritional status and sarcopenia, independently of BMI^(42)^.

**Table 1.** COVID-19 symptoms potentially related to weight loss and cachexia (Mean values and standard deviations; medians and interquartile ranges (IQR))

Study	Country	Study design	Number of patients	Age (years)			Sex	COVID-19 symptoms potentially related to weight loss and cachexia
				Mean		SD		
Chen *et al.*^(2)^	China	Retrospective cohort	99	55·5		13·1	Male (68 %); female (32 %)	Decreased Hb
								Decreased albumin
								Increased C-reactive protein
								Increased IL-6
								Fever
								Muscle ache
								Headache
								Diarrhoea
								Nausea and vomiting
Huang *et al*.^(33)^	China	Prospective cohort	41				Male (73 %); female (27 %)	Decreased albumin
Median					49			Fever
IQR					41–58			Myalgia or fatigue
								Headache
								Diarrhoea
Li *et al.*^(20)^	China	Cross-sectional	182	68·5		8·8	Female (64 %); male (36 %)	Decreased Hb
								Decreased albumin
								Increased C-reactive protein
Jin *et al.*^(28)^	China	Retrospective cohort	74	46·14		14·2	Female (50 %); male (50 %)	Increased C-reactive protein
								Fever
								Fatigue
								Muscle ache
								Headache
								Diarrhoea
								Nausea and vomiting
Wan *et al.*^(34)^	China	Case series	135				Male (53 %); female (47 %)	Decreased albumin
Median					47			Increased C-reactive protein
IQR					36–55			Fever
								Myalgia or fatigue
								Loss of appetite
								Diarrhoea
Brill *et al.*^(35)^	UK	Retrospective cohort	450				Male (60 %); female (40 %)	Increased C-reactive protein
Median					72			
IQR					56–83			
Liu *et al.*^(36)^	China	Retrospective cohort	137				Female (55·5 %); male (44·5 %)	Increased C-reactive protein
Median					57			Fever
IQR					20–83			Myalgia or fatigue
								Headache
								Diarrhoea
Xiong *et al.*^(37)^	China	Retrospective cohort	42	49·5		14·1	Male (60 %); female (40 %)	Increased C-reactive protein
								Fever
								Fatigue
								Diarrhoea
Li *et al.*^(38)^	China	Retrospective cohort	90	45·5		12·3	Male (53 %); female (47 %)	Increased C-reactive protein
								Fever
								Myalgia
								Headache
								Abdominal pain/diarrhoea

Based on available clinical observations, it is evident that although people of all ages can become infected and present weight loss, elderly people with low immunity and patients with chronic diseases have a worse prognosis and have a higher risk of cachexia. So, it is important to highlight the importance of weight loss monitoring and nutritional status vigilance for these patients. Nutritional therapy should be regarded as first-line treatment and implemented into standard of practice^(25)^, representing primary guarantee for promoting disease recovery.

## Interrelations between prolonged hospitalisation, weight loss and risk of cachexia

SARS-CoV-2 manifestation and COVID-19 disease might be asymptomatic or present moderate to severe symptoms. In moderate and more severe cases, hospitalisation is necessary and complications can include acute respiratory distress syndrome, acute cardiac complications, multiple organ dysfunction syndrome, septic shock and death^(43)^. These complications have been described as the cytokine storm, in which viral replication triggers an abnormally strong release of cytokines and other immune-related stimuli, resulting in inflammation^(14)^ and weight loss^(20)^.

Identification of risk and presence of malnutrition should be an early step in the general assessment of all patients, with regard to more at-risk categories including older adults and individuals suffering from chronic and acute disease conditions, as COVID-19. Malnutrition can be defined as any nutritional imbalance which can happen both in underweight and overweight individuals^(44)^. This imbalance can be originated from different conditions such as insufficient nutrient intake, higher energy requirements, impaired absorption and/or changes in nutrient utilisation and transport^(45)^. In addition, malnourished patients have higher risks of poorer outcomes such as longer length of hospital stay, muscle wasting, postoperative complications, depression of the immune system and mortality^(44)^.

**Fig. 1.**
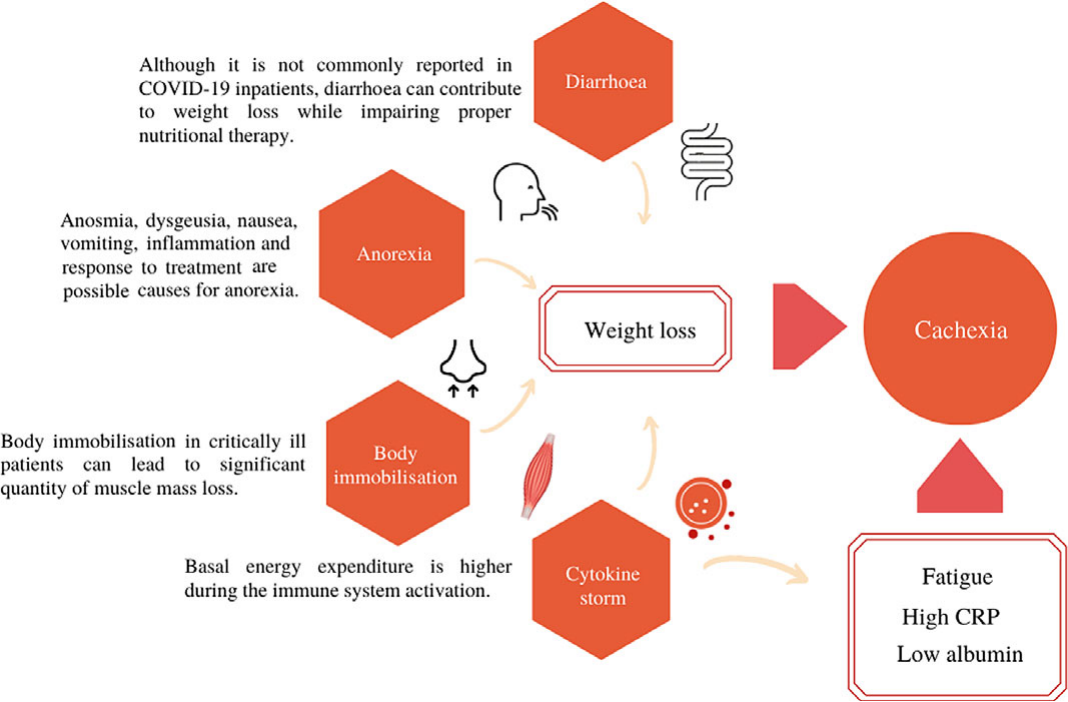
Symptoms in COVID-19 inpatients related to the onset of cachexia. CRP, C-reactive protein.

It is well known that a healthy nutritional status is important for immune system support and to prevent severe infections^(14)^. Nevertheless, the nutritional status of people with viral infections was not described to be a risk factor in the emergence of viral diseases which could be due to a lack of data, making it even more important to be reported^(29)^. In COVID-19 patients, these challenges appear to result from the direct effects of the SARS-CoV-2 virus on the gastrointestinal tract and are compounded by the elevated sedation required for this patient^(46)^. Also, the elevated doses of sedatives and opioids required to facilitate mechanical ventilation in patients with COVID-19 can contribute to intestinal dysmotility and weight loss^(25)^ which may lead to cachexia. Fig. 1 illustrates, in a didactic way, how the frequent manifestations presented by patients with COVID-19 can induce weight loss and, consequently, cachexia.

Cachexia is a complex metabolic syndrome associated with underlying illness and characterised by loss of muscle with or without loss of fat mass^(47)^. Cachexia can also be defined as ‘weight loss in the presence of illness, combined with three or more of the following five criteria: decreased handgrip strength, fatigue, anorexia, low fatty free mass index (FFMI) or abnormal biochemistry (high C-reactive protein, low hemoglobin or low albumin)’^(48)^. Many of these factors that must be associated with weight loss for the diagnosis of cachexia are frequent symptoms in COVID-19 patients. Table 2 shows the interrelations between the signs and symptoms present in cachexia syndrome and COVID-19 patients.

**Table 2.** Interrelations between the signs and symptoms present in cachexia and COVID-19 patients

Cachexia and its relation to COVID-19		
Definition of cachexia^(48)^	Weight loss of at least 5 % in 3 months (or BMI < 20 kg/m^2^) + (minimum three items)	(1) Decreased muscle strength (2) Fatigue* (3) Anorexia* (4) Decreased fat-free mass index (5) Decreased biochemistry levels (high C-reactive protein*, low Hb, or low albumin*)

* The signs and symptoms usually observed in COVID-19 patients.

To date, data regarding decreased muscle strength and low fat-free mass index were not reported in recent studies in patients with COVID-19. However, they could be further explored using a hand dynamometer for assessing muscle strength and computed tomography (CT) acquired as part of standard COVID-19 to the determination of body composition and diagnosis of muscle mass reduction in these patients. The use of imaging diagnostics for body composition makes maximal use of existing information and could help to recognise and treat patients at increased risk of wasting with targeted pathways. In fact, some studies have already used CT images to describe the body composition of patients with COVID-19^(49–51)^, showing that an inadequate body composition increases the risk of complications from the disease. In general, cross-sectional analysis of single CT images, typically landmarked at the 3rd lumbar vertebra (L3), is conducted^(52,53)^. It is possible that some CT images of patients with COVID-19 do not include the L3 region, but it is still possible to estimate body composition in other regions of the abdomen, as the thoracic area^(54)^. The information obtained through CT analysis, quantifying the patient’s muscle mass, can improve the care of the critically ill patient, establishing early conducts for the cachexia management.

Fatigue is a debilitating symptom commonly reported by COVID-19 patients^(55)^. Its aetiology and pathophysiology are not well understood yet, still it is suggested that both central and peripheral mechanisms are involved in the physical expression of fatigue^(56)^. Up to date, the exact duration of this symptom is not reported by the studies as well, it is still unclear for how long patients can experience COVID-19-related fatigue. This could be due to the ‘cytokine storm’ leading to inflammation and anorexia, both responsible for muscle loss, weakness and fatigue. Also, infections contribute to higher basal energy expenditure during the immune system activation in which fever is a common symptom^(57)^. Still, studies regarding the aetiology of fatigue, its duration and associated diseases in COVID-19 patients have to be better explored.

Anorexia has also been reported as a common symptom in recent meta-analyses^(58–60)^. It is a complex and multifactorial symptom which can occur in the presence of acute inflammatory disease adverse reactions to treatment, depression, altered liver function and hypoxia^(61,62)^. Diarrhoea, nausea and vomiting were less frequent gastrointestinal symptoms than anorexia^(60)^. It is important to mention the presence of dysgeusia and anosmia of COVID-19 as possible contributors of anorexia.

There is expressive evidence of low albumin, other inflammation biomarkers, anorexia and fatigue as common symptoms in patients with COVID-19. Observational studies show low albumin in severe cases^(34,63)^. A meta-analysis has reported that severe cases of COVID-19 were associated with hypoalbuminaemia, but the relation between the disease and the biomarker is still unclear^(63)^. Albumin circulating levels should not be considered as a nutritional marker in patients with inflammatory response^(21,42)^, but this biochemical marker has good relation with muscle mass and high-sensitive C-reactive protein.

High high-sensitive C-reactive protein usually observed in COVID-19 patients, confirming its viral aetiology as well as a biomarker of cachexia, is commonly altered in patients as a result of the ‘cytokine storm’ which increases the severity of COVID-19^(34,55,64,65)^. The rapid recruitment of neutrophils and macrophages causes an exacerbated reaction to infections producing pro-inflammatory cytokines and modifying the fragile balance between the host-damaging reaction and a controlled immune response^(66)^. The acute phase protein is a sensitive biomarker in which the up-regulated synthesis occurs in tissue damage, malignant neoplasia, infections and inflammation^(67)^.

Low Hb is a biomarker present in the cachexia syndrome, and it was reported in COVID-19 patients in a few studies^(2,20)^. However, it may be worth evaluating the role of Hb in the pathophysiology of COVID-19 and investigating its relation to the illness and adverse outcomes. Sotoudeh *et al*.^(68)^ reported that the mortality rate of COVID-19 patients is lower in countries with a higher prevalence of haemoglobinopathies. This finding does not prove the direct association, and the observed finding can be due to other confounding variables or poor patient detection in tropical countries. A systematic review shows that six studies described low Hb in patients with COVID-19, while four studies found that all patients had Hb within the reference interval^(69)^. However, a limitation for this alteration is that most studies reported laboratory analysis from the patients at admission.

Few studies reported how the severity of COVID-19 leads to longer LOS compared with milder cases^(70,71)^. Bhatraju *et al*. showed that the median LOS among survivors of critically ill COVID-19 patients was 17 d (interquartile range, 16–23), and the median length of intensive care unit (ICU) stay among survivors was 14 d (interquartile range, 4–17). Prolonged length of hospital stay may be accompanied by loss in weight and muscle mass^(72)^, leading to cachexia^(73,74)^. LOS is usually lacking information in COVID-19 reports, and it should be given more attention considering it is an adverse outcome. Thus, nutrition therapy in patients with cachexia can be effective, and it was shown to significantly reduce LOS^(75)^.

## Prevention and treatment of COVID-19 cachectic patients through diet

Although most critically ill patients with COVID-19 are overweight or obese^(15)^, they are often sick at home for days to weeks prior to being admitted to the hospital, thus increasing their likelihood of being malnourished upon presentation^(46)^.

Malnutrition differs from cachexia due to its complex nature which combines an underlying disease, metabolic alterations and sometimes a reduced nutrient availability which might play an important role on the onset of the syndrome^(21)^, while malnutrition is not described as a syndrome but an altered state of nutrition due to different causes. Therefore, cachexia must be distinguished from other causes of muscle loss. The clinical consequences of cachexia depend as much on the weight loss as on the systemic inflammation, which accompany the development of cachexia^(76)^. Muscle, bone and fat tissue loss are also reported in cachexia^(77,78)^. Prolonged length of hospital stay is another situation that may be accompanied by loss in weight and muscle mass^(72)^. Although nutrition therapy is essential to promote better outcomes for patients, it is still often overlooked in clinical practice^(44)^.

Established interventions to treat cachexia are available for specific underlying diseases, for example, progestogens^(79)^, corticosteroids and other different approaches including, for example, myostatin antagonists, ghrelin agonists, selective androgen receptor antagonists were described^(80)^. To impair muscle mass loss, exercises should be included in the treatment even in the individual with advanced cachexia to reduce muscle mass and physical function loss^(81)^.

The European Society for Clinical Nutrition and Metabolism and other authors proposed general nutritional recommendations for all stages of COVID-19^(25,26,82)^. However, there is no data in the literature regarding nutritional recommendations for the prevention and treatment of cachexia in COVID-19 patients. The European Society for Clinical Nutrition and Metabolism document aims to provide concise statements from experts and practical guidelines for the nutritional management of patients with COVID-19, whether for adults with polymorbidity or those in the ICU setting, which are independent factors associated with malnutrition and negative outcomes^(82)^. In summary, this reference guides an energy supply between 27 and 30 kcal/kg per d (113 and 126 kJ/kg per d) (according to the nutritional status), and a protein supply of 1 g/kg.

A recent review summarised the clinical Chinese observations and compared them with the references brought by European Society for Clinical Nutrition and Metabolism’s guidelines^(26)^. Zhang & Liu^(29)^ proposed several treatment options (including nutritional interventions) for the novel coronavirus, based on a review of the current literature^(41)^. There are macronutrient recommendations for patients with COVID-19 pointed out in those studies. For lipid and carbohydrate needs, it is possible to consider an energy ratio from fat and carbohydrates from total estimated energy in a percentual distribution of 30:70 for subjects with no respiratory deficiency to 50:50 in ventilated patients^(82)^. It can also be considered 2 g/kg per d, not exceeding 150 g/d for carbohydrate and 1·5 g/kg per d for fat in critically ill patients^(25)^.

In COVID-19 intubated and ventilated ICU patients, enteral nutrition should be started. European Society for Clinical Nutrition and Metabolism suggests that in the early phase of the acute illness, a hypoenergetic nutrition of 20 kcal/kg per d (84 kJ/kg per d) should be administered (not exceeding 70 % of estimated energy) with increments up to 80–100 % after the third day. During critical illness, 1·3 g/kg protein equivalents per d can be delivered progressively. The use of enteral *n*-3 fatty acids may improve oxygenation despite strong evidence still not being available^(82,83)^. Up to date, the studies described in literature discuss nutritional management in ICU patients with cachexia due to other causes and report the importance of using specific nutrients, especially amino acids, to minimise excessive muscle mass loss^(83)^.

Leucine has been shown to be a potent stimulator of protein synthesis via the mammalian target of rapamycin complex pathway. It has recently been shown to be effective in elderly subjects with sarcopenia^(84)^. It may be extrapolated for COVID-19 patients with cachexia, but this use needs more studies. Arginine and glutamine are nonessential amino acids that are widely discussed in the literature for critically ill patients, but their role in muscular recovery is still unclear^(83)^. A recent report on nutritional support in patients with COVID-19 reaffirmed the use of arginine for immunity and healing, as well as the role of glutamine in preserving intestinal function, but not in muscle recovery in either nutrient^(25)^.

*n*-3 PUFA, *β*-hydroxy-*β*-methylbutyrate (HMB) and l-carnitine^(76,85,86)^ are some other target nutrient suggestions for the recovery process. *n*-3 PUFA have shown to optimise tissue recovery^(76)^. A systematic review with HMB concluded that this amino acid metabolite attenuates exercise-induced muscle damage and enhances muscle hypertrophy and strength^(86)^. l-Carnitine supplementation has protective effects on several mechanisms of muscle loss, improving protein synthesis^(85)^.

It is also important to monitor and assess micronutrient levels and supplement accordingly. Overall, low levels or intakes of micronutrients such as vitamins A, E, D, B_6_ and B_12_, Zn and Se have been associated with adverse clinical outcomes during viral infections^(25,82)^. For the assessment of micronutrients in COVID-19 patients, vitamins A and D, vitamin B, vitamin C, *n*-3 PUFA, as well as Se, Zn and Fe should also be considered^(32,41).^

For cachectic patients, no guidelines exist for its prevention or treatment. Appetite stimulants, such as megestrol acetate and glucocorticoids, have been shown to increase appetite and weight^(75)^. However, in the acute phase of the COVID-19 disease and in the presence of intubation, these drugs are considered futile. Nowadays, clinicians should consider personalised nutritional treatment as target to prevent and treat cachexia for each patient. These interventions would include, as possible according to the general state of a patient, nutritional counselling, assessing and treating symptoms that have an impact on energetic intake, and a rational combination of pharmacological approaches directed at underlying pathophysiology^(87)^. Laboratory measurements of nutritional status, such as albumin levels, may be useful in certain cases. Table 3 summarises the purpose of nutritional recommendations for COVID-19 patients with cachexia during critical illness and/or admitted in a critical care unit.

**Table 3.** Prevention and treatment of malnutrition and cachexia in COVID-19 patients

Energy	Protein	Other recommendations
Pre-intubation and/or oral diet		
25–30 kcal/kg (105–126 kJ/kg)	1·0 g/kg	It should be individually adjusted regarding nutritional status, physical activity level, disease status and tolerance
Intubation and/or enteral diet		
21–30 kcal/kg (88–126 kJ/kg) (eutrophic)	1·2–2·5 g/kg	For patients with gastric residuals above 500 ml, the ESPEN guidelines^(83)^ recommend placement of a post-pyloric feeding tube as soon as possible
11–25 kcal/kg (46–105 kJ/kg) (obese)		
<20 kcal/kg (<84 kJ/kg) (parenteral nutrition)	1·3 g/kg (parenteral nutrition)	
Rehabilitation		
25–30 kcal/kg (105–126 kJ/kg)	0·8–1·2 g/kg	Leucine: 3 %/d
		l-Carnitine: 2–4 g/d orally
		*n*-3 (EPA): 2 g/d
		HMB: 1·5–3 g/d (along with exercise stimulation)

ESPEN, European Society for Clinical Nutrition and Metabolism; HMB, *β*-hydroxy-*β*-methylbutyrate.

The risk of refeeding syndrome in critically ill patients with COVID-19 should be monitored, as cachexia increases nutritional deficits^(46)^. In addition, electrolyte disturbances increase the risk of refeeding syndrome and contribute to arrhythmias and hemodynamic instability. So far, we are not aware of specific recommendations for patients after extubation and in the state of rehabilitation, but it is important to pay attention to the risk of dysphagia and swallowing assessment procedures should be applied to assess the possibility of implementing texture-adapted food in this condition^(82)^.

## Conclusions

Governments of countries around the globe should be dealing with the possibility of prolonged hospitalisation of COVID-19 patients and its enormous strains on the healthcare system. The deterioration of nutritional status, and consequently cachexia, increases the risk of mortality and needs to be treated with attention as other complications. Ensuring adequate nutrition in patients with COVID-19 who presented cachexia or associated symptoms has proven to be challenging due to intestinal alterations and inflammatory profile which complicate nutritional management. There is, however, little hard evidence of nutritional health approaches in assisting COVID-19 treatment or its management including cachexia. Nevertheless, COVID-19 is still a recently discovered disease, measures regarding maintenance of nutritional status and prevention of cachexia in hospitalised patients should be better explored for effective nutritional interventions in the future.
